# Mortality, Temporary Sterilization, and Maternal Effects of Sublethal Heat in Bed Bugs

**DOI:** 10.1371/journal.pone.0127555

**Published:** 2015-05-21

**Authors:** Bjørn Arne Rukke, Anders Aak, Kristin Skarsfjord Edgar

**Affiliations:** Norwegian Institute of Public Health, Department of Pest Control, Lovisenberggata 8, PO Box 4404, Nydalen, NO-0456, Oslo, Norway; University of Cincinnati, UNITED STATES

## Abstract

Adult bed bugs were exposed to the sublethal temperatures 34.0°C, 35.5°C, 37.0°C, 38.5°C, or 40.0°C for 3, 6, or 9 days. The two uppermost temperatures induced 100% mortality within 9 and 2 days, respectively, whereas 34.0°C had no observable effect. The intermediate temperatures interacted with time to induce a limited level of mortality but had distinct effects on fecundity, reflected by decreases in the number of eggs produced and hatching success. Adult fecundity remained low for up to 40 days after heat exposure, and the time until fertility was restored correlated with the temperature-sum experienced during heat exposure. Three or 6 days of parental exposure to 38.5°C significantly lowered their offspring’s feeding and moulting ability, which consequently led to a failure to continue beyond the third instar. Eggs that were deposited at 22.0°C before being exposed to 37.0°C for 3 or 6 days died, whereas eggs that were exposed to lower temperatures were not significantly affected. Eggs that were deposited during heat treatment exhibited high levels of mortality also at 34.0°C and 35.5°C. The observed negative effects of temperatures between 34.0°C and 40.0°C may be utilized in pest management, and sublethal temperature exposure ought to be further investigated as an additional tool to decimate or potentially eradicate bed bug populations. The effect of parental heat exposure on progeny demonstrates the importance of including maternal considerations when studying bed bug environmental stress reactions.

## Introduction

Bed bugs, *Cimex lectularius* (Hemiptera: Cimicidae), have made a considerable comeback as a nuisance pest over the last 15 years [1]. These ectoparasites are blood-feeders on humans and induce both negative physical and mental reactions [2,3]. Recently, bed bugs have been shown to harbour *Trypanosoma cruzi* to potentially transmit Chagas disease [4]. Their resurgence is based on pesticide resistance, increased globalization, and insufficient knowledge of necessary actions to prevent and control infestations [5]. Several methods are currently used to control bed bugs, and an integrated pest management strategy is considered necessary in the majority of cases [2,6]. Heat treatment is an important management approach that can be combined with most other traditional control methods. To control bed bugs with heat, high temperatures are utilized through the localized heat treatment of objects, surfaces, and harbourages [7–9] or as whole-compartment management using larger heat generator systems [10]. The goal for all bed bug treatments is to kill bed bugs instantly or within a few hours by exposing them to high temperatures. The use of sublethal temperatures for longer periods of time has not yet been considered an option against bed bugs, although such thermal stress may induce mortality and reproductive abnormalities [11–13].

Physiological mechanisms that improve tolerance to fluctuating abiotic conditions are essential in an organism’s biology and ensure proper function in a variable ecosystem [14,15]. At high temperatures, death, behavioural impairment, or developmental instability may in insects result from such factors as denaturation of proteins, accumulation of toxic products, DNA damage, pH changes, loss of membrane function, nutrient deprivation, or desiccation [15,16]. Heat stress can be countered behaviourally by moving to more favourable microclimatic conditions or physiologically by heat tolerance through acclimation or rapid heat hardening [15]. Improvements in heat tolerance often involve heat shock proteins that act as chaperones to protect other proteins from denaturing, or that bind to the surface of an aggregate of proteins and promote their dissolution causing the proteins to refold [17,18]. However, sustained high levels of these proteins may also induce detrimental effects by indirectly affecting development, fecundity, and survival [19–21]. Bed bugs have the capacity to handle desiccation stress, and rapid changes in body temperature and water content during blood feeding [22–25]. On the other hand, as they originate from stable temperature conditions in temperate bat caves [26,27], adaptions to persistently high temperatures are unlikely. This potential lack of extensive temperature tolerance may be utilized to control bed bugs because human-made habitats can be strictly temperature regulated.

No bed bug study has yet investigated both the stage- and generation-dependent influence of heat stress to reveal the potentially detrimental effects of reduced survival and fecundity on the population. The present study tested how sublethal temperatures affect bed bugs and their offspring. We focused on adult survival in combination with fecundity measures and progeny success. The reported findings suggest a broader role for heat treatment in bed bug control by adding sublethal temperatures as a potential component in future management approaches.

## Material and Methods

### Bed bug cultures

Bed bugs in stock cultures were sampled from two hotels in Oslo, Norway, in 2009. Permission to sample bed bugs was given by the hotel owners. The initial population consisted of approximately 40 adults from each hotel. The stock cultures were maintained in a 16 h/8 h light/dark cycle at 22°C and 65% relative humidity. All of the experimental animals were fed heated human blood through a Parafilm membrane [28]. Small samples of blood were donated voluntary by two of the authors, after written consent. Approval from an institutional review board or ethics committee was not required. We used a mixture of approximately equal amounts of animals from each of the two stock cultures. To produce standardized experimental animals of equal age, fifth-instar nymphs were selected from the stock cultures, transferred to a new box, and fed. Newly hatched adults emerged after 10–14 days. These adults were fed, and fully engorged bed bugs were rested 1 day and allowed to mate, before distribution into the experimental units.

### Test chambers and experimental units

#### Abiotic conditions

The experiments were performed in climate chambers (Sanyo—MLR-351H, Medinor ASA, Oslo, Norway) with a 16 h/8 h light/dark cycle and 65% (64.5% ± 0.08% [SE]) relative humidity. Temperatures were maintained at 22.0°C (22.1°C ± 0.02°C), 34.0°C (33.7°C ± 0.00°C), 35.5°C (35.4°C ± 0.01°C), 37.0°C (36.9°C ± 0.00°C), 38.5°C (38.4°C ± 0.01°C), or 40.0°C (39.6°C ± 0.02°C) according to the needs of the experiments. We used desiccators (VWR Desiccator 250, VWR, Oslo, Norway) that contained a humidity absorber (Damp Eater Torrbollen 500 g, Säljtema, Linköping) to attain 5% (6.9% ± 0.02%) relative humidity in one of experiments. A temperature of 22.0°C combined with 65% relative humidity was considered our standard climatic conditions and hereafter denoted only as room temperature. Unless otherwise stated, each climate chamber was held at 65% relative humidity.

#### Bed bug boxes

In all the experiments, animals were placed in140 ml polyethylene boxes (VWR straight sample container, VWR, Oslo, Norway) that contained a 2 × 2 cm piece of filter paper (VWR qualitative filter paper, VWR, Oslo, Norway). The plastic lids of the boxes had circular openings (40 mm diameter) into which metal mesh screens (0.25 mm openings; Burmeister AS, Oslo, Norway) were inserted to facilitate the passage of air and to allow bed bugs to feed through it. To ease recording of bed bug survival and feeding, each polyethylene box contained three males and three females in the experiments with adults. Successful feeding was scored if the abdomen of the bed bug was extended. For cohort studies, each box initially contained 10 nymphs.

### Experimental design

#### Heat tolerance screening

Temperatures of 34.0°C, 35.5°C, 37.0°C, 38.5°C, and 40.0°C were combined with either 5% or 65% relative humidity. Thirty adult bed bugs were tested at each of the 10 temperature-humidity combinations. The control was maintained at room temperature. Individual mortality, the number of eggs, and the number of emerged nymphs were recorded after 3, 6, 12, and 24 h and daily thereafter for the next 8 days of elevated temperature. On day 9, all of the surviving adults were moved to room temperature, fed, and relocated into one box per treatment. Eggs that were laid during the 9 days of heat treatment were maintained at room temperature and allowed to hatch in the original boxes for another 14 days. After 14 days, the adults were again moved to a new box, and survival, egg production, and emerged nymphs were recorded. The adults were not fed at this point. This procedure was repeated once more to provide a total of 6 weeks of recording after heat treatment and feeding.

#### Sublethal heat treatment of adults

Based on the screening results, we exposed the adults to 38.5°C or 37.0°C for 1, 3, or 6 days and 35.5°C for 3 or 6 days. Each of the eight temperature-day combinations was tested on 60 adult bed bugs and compared with 60 control bed bugs that were kept at room temperature. After the assigned time of temperature exposure, the boxes were moved to room temperature, and mortality was recorded on day 9. Dead bed bugs were removed, and survivors were relocated to obtain as many boxes with six living bed bugs as possible. For 8 weeks, adults were moved to new boxes every 7 days, and survival, egg production, and emerged nymphs were recorded. Additionally, on day 9 and every 14 days thereafter, the adults were fed, and the proportion that took a blood meal was recorded.

#### Offspring effects

Fifty newly hatched first-instar nymphs, which originated from parents that experienced high temperatures 7 weeks earlier (35.5°C or 37.0°C for 6 days or 38.5°C for 1, 3, or 6 days), were followed for up to 16 weeks. Additionally, 50 newly hatched first-instar nymphs with parents that had been kept at room temperature were used as controls. All 300 nymphs that originated from the six different treatments showed a normal appearance and movement when transferred to the experimental boxes. They were fed every 14 days, and mortality and the number of nymphs that fed were recorded. After four feeding events, the success of each cohort was evaluated by counting exuviae. Each series was terminated upon the appearance of the first adult or when all 50 individuals were dead.

#### Heat treatment of eggs

Untreated, newly emerged adults were fed and allowed to lay eggs on filter paper in the experimental boxes. After 3 days, they were transferred to new boxes to lay eggs for three additional days. After these 6 days of egg laying, the adults were killed. As soon as the adults were removed, the boxes that contained only the eggs and filter paper were assigned to room temperature (control), 34.0°C, 35.5°C, or 37.0°C for 3 or 6 days. The boxes contained 3–18 eggs each, providing 626 eggs that were distributed between the seven treatments. The eggs were 1–3 days old when exposed to heat stress. After heat exposure, the boxes were returned to room temperature for 14 days before hatching success was scored.

### Statistical analyses

The data were analysed using SigmaPlot 12.3 (Systat Software, San Jose, CA, USA) and JMP Pro 11.1.1 (SAS institute, Cary, NC, USA). The data were checked for normality, and multiple comparisons were performed using analysis of variance (ANOVA). Pairwise comparisons were performed using *t*-tests. Differences between multiple comparisons were identified using Dunnett’s test for comparisons with a control group. The level of significance was set to 0.05. If tests for normality failed, then we used the nonparametric alternatives Wilcoxon signed-rank and Kruskal-Wallis ANOVA with Dunn’s pairwise comparisons. We used the Kaplan-Meier product limit method with the log-rank test between groups to analyse survival. To identify the time-temperature effect on fecundity, linear regression was used with the day-degrees experienced above a bed bug optimum temperature of 28°C [29] as the predictor variable and the time until fertility recovery as the response.

## Results

### Heat tolerance screening

**Fig 1:**
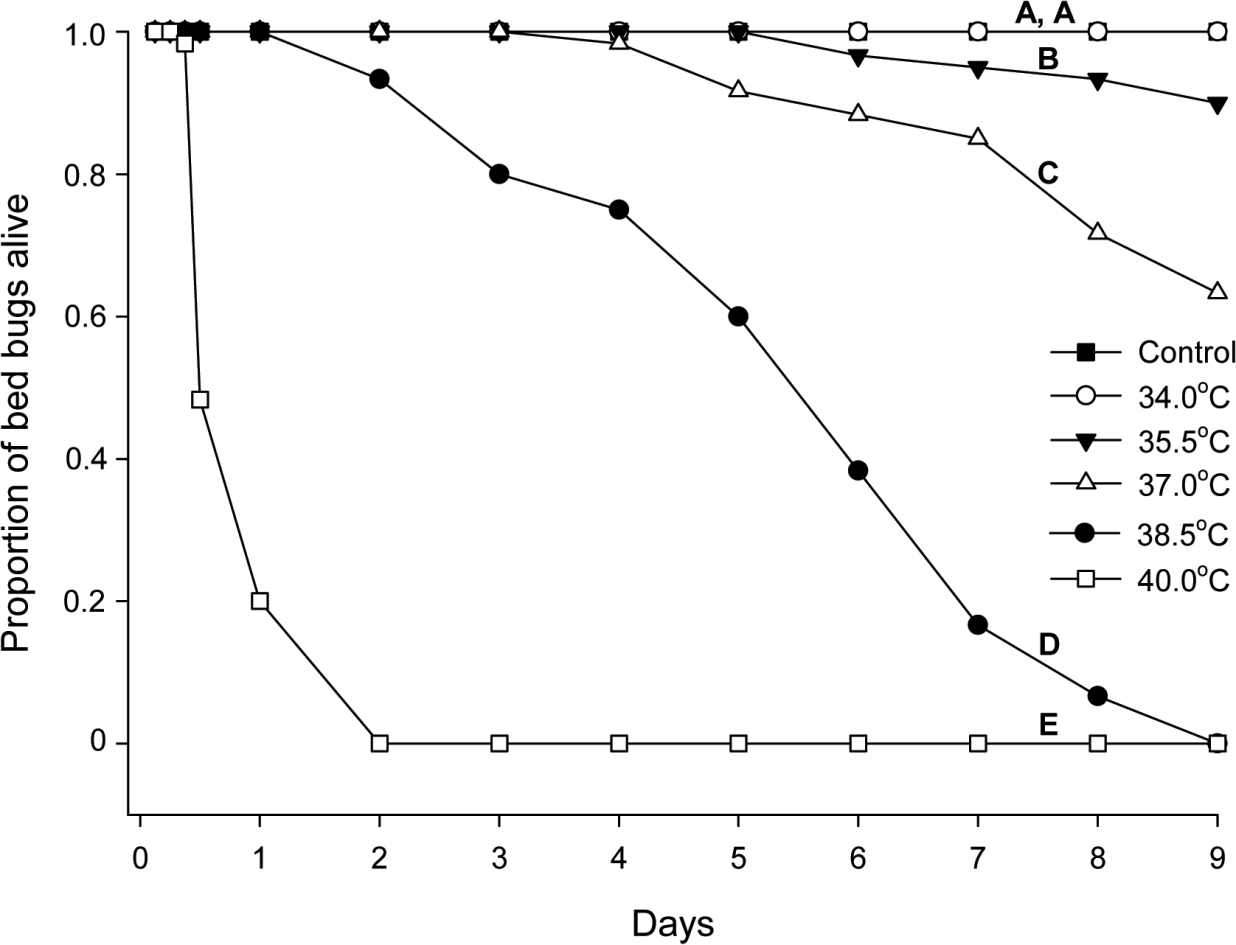
Survival of heat treated adult *Cimex lectularius*. The proportion of adults alive during different heat treatments for 9 days is shown. Control = 22.0°C. Different letters denote significant differences in survival between treatments (*p* < 0.05).

No differences in survival were found between sexes when exposed to 22.0°C, 34.0°C, 35.5°C, 37.0°C, 38.5°C, and 40.0°C (Kaplan-Meier; females *vs*. males at each temperature and humidity combination, *p* > 0.1 in all tests). High humidity increased mortality only at 40.0°C and 38.5°C (Kaplan-Meier; 65% *vs*. 5% relative humidity at 40.0°C: *χ* ^*2*^ = 10.24, df = 1, *p* = 0.001; and at 38.5°C: *χ* ^*2*^ = 8.01, df = 1, *p* = 0.005). Mortality significantly increased with increasing temperatures (Kaplan-Meier; 34.0°C *vs*. 35.5°C: *χ* ^*2*^ = 6.26, df = 1, *p* = 0.02, with lower *p* values for the remaining consecutive pairwise comparisons; Fig 1). Dead bed bugs also appeared first at the highest temperatures, and 38.5°C and 40.0°C induced 100% mortality within 9 and 2 days, respectively. Egg production and hatching success decreased with increasing temperature (Table 1). Even at 34.0°C, hatching success was only 50% compared with the control. This fecundity remained low at 37.0°C, but it was restored at 34.0°C and 35.5°C after 24–37 days. No viable eggs were produced 28 days after the last feeding.

**Table 1:** Eggs hatched and laid per female *Cimex lectularius*.

		During Treatment			After treatment						Cumulative
.		Day 1–9			Day 10–23		Day 24–37		Day 38–51		Day 1–51
		Hatching success	Eggs per female		Hatching success	Eggs per female	Hatching success	Eggs per female	Hatching success	Eggs per female	Hatching success
Control	●	98.4%	4.1	●	99.2%	8.3	93.3%	1.0	-	0.0	98.5
34.0°C	●	54.4%	3.7	●	43.1%	8.7	73.5%	1.1	-	0.0	48.5
35.5°C	●	0.5%	6.1	●	19.8%	8.9	81.8%	1.6	-	0.0	18.1
37.0°C	●	0.0%	1.2	●	6.7%	0.8	0%	1.3	0%	0.2	1.5
38.5°C	●	0.0%	0.1	-	-		-		-		0
40.0°C	●	0.0%	0.0	-	-		-		-		0

Percentage of eggs hatched and eggs laid per female exposed to different temperatures for 9 days and then evaluated 10–23, 24–37, and 38–51 days after heat treatment while maintained at 22°C. The last column denotes the average percentage of eggs hatched over all periods.

● = feeding event.

### Sublethal heat treatment of adults

**Fig 2:**
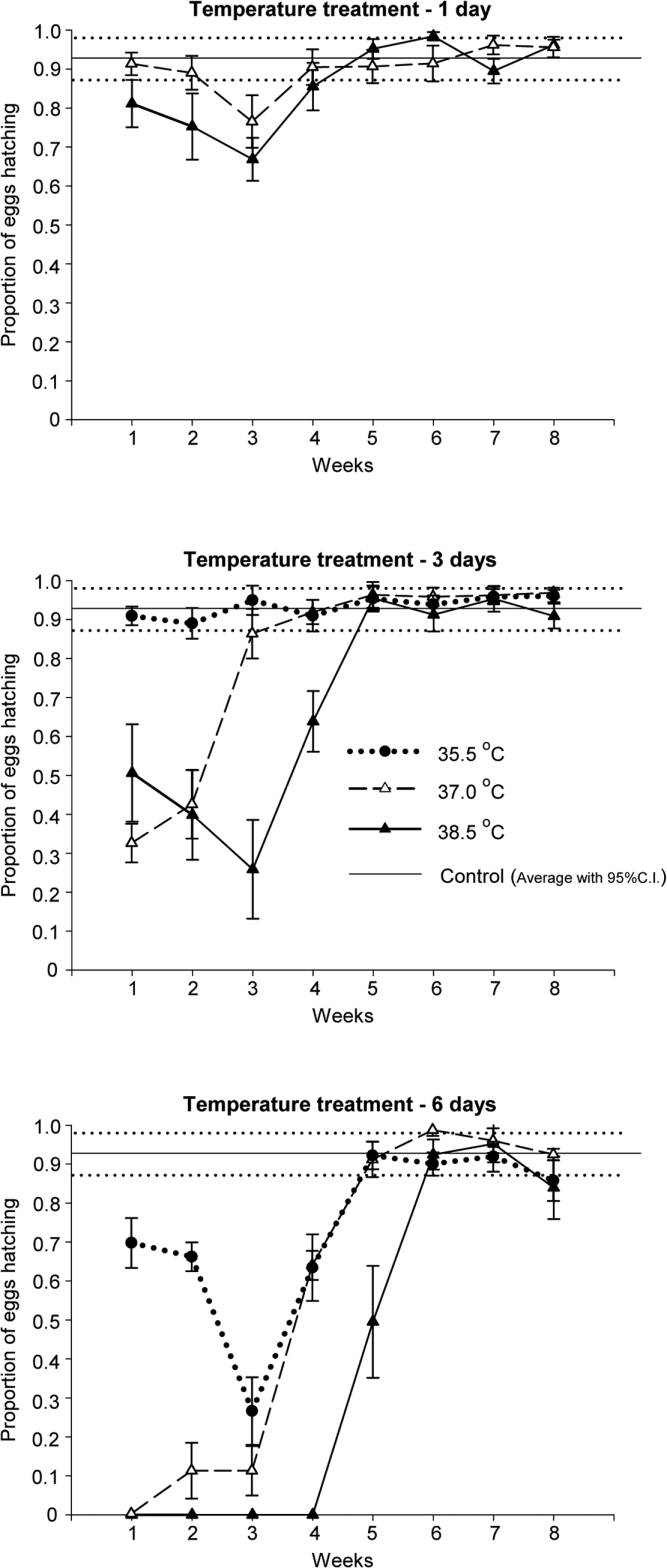
Restoration of fecundity in heat treated adult *Cimex lectularius*. The restoration of fecundity in adults after 1, 3 or 6 days of heat treatment is reflected by egg hatching success (mean ± SE) in successive weeks after day 9 of the experiment. Control = 22.0°C. After heat exposure, the adults were kept at 22.0°C and fed every 14 days.

**Fig 3:**
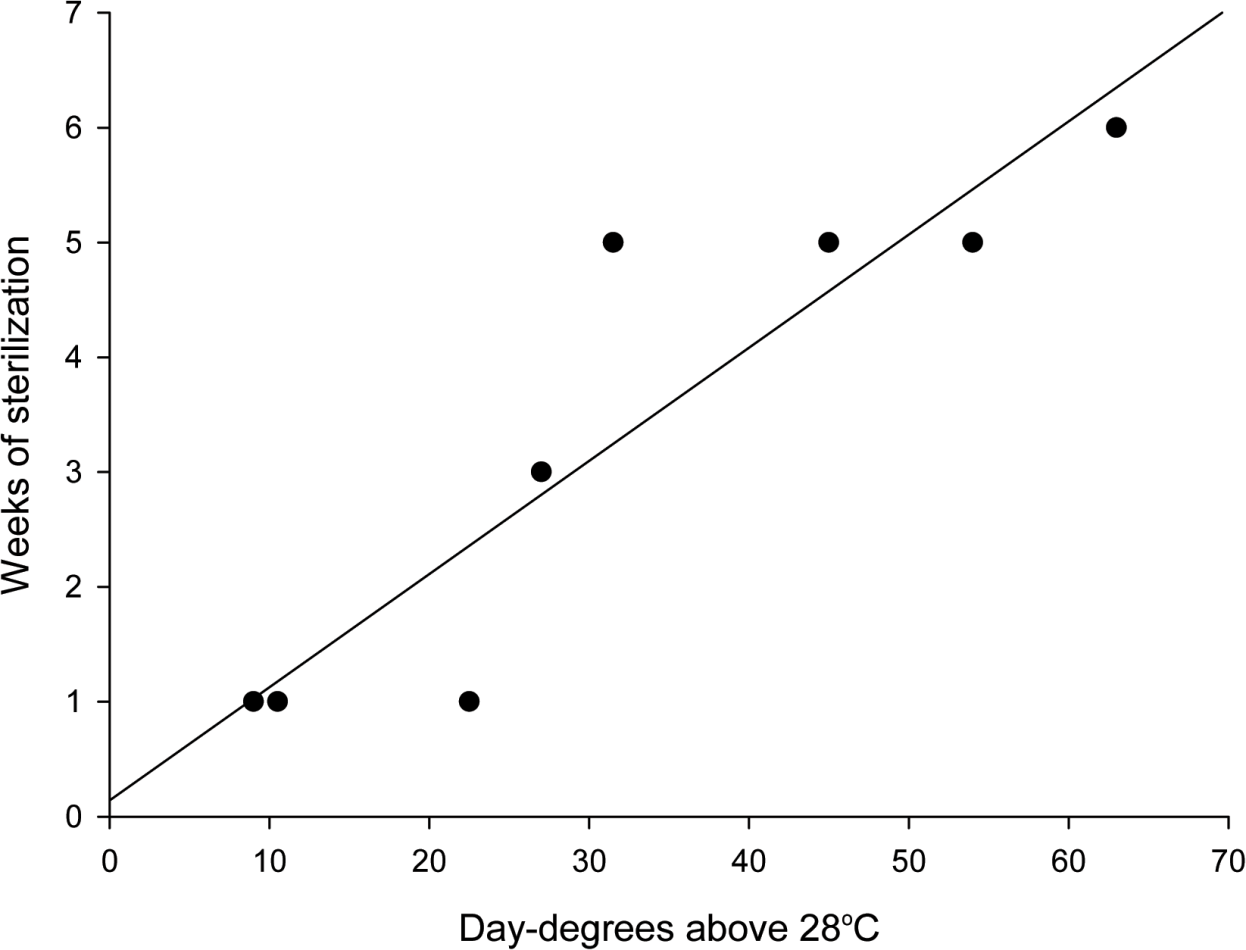
Day degrees experienced and recovered fertility in heat treated adult *Cimex lectularius*. The correlation between day-degrees above the optimum developmental temperature for *Cimex lectularius* (28°C) experienced by adults under heat treatment and the time until their fertility recovered is described. Line equation: weeks = 0.142 + (0.099 × day degrees).

Egg hatching success was not influenced by 1-day temperature treatments, but prolonged heat exposure of the adults temporarily impaired hatching success (Fig 2, summarized statistics in Table 2). Three days of exposure to 38.5°C resulted in fecundity that was significantly lower than the control, whereas 3 days of exposure to 35.5°C and 37.0°C had no long-term effect. Six days of exposure significantly reduced fecundity at all three temperatures compared with the control. Both the length of treatment and temperature influenced the time until fecundity recovered. This combined effect was best described by a correlation between the day-degrees experienced above the bed bug optimum temperature and the time until fertility recovered (Linear Regression; *R* ^*2*^ = 0.827, *p* = 0.002; Fig 3).

**Table 2:** Comparisons of fertility of female *Cimex lectularius*.

	1 day	3 days	6 days
Relative to control:			
Room temperature *vs*. 35.5°C	x	ns	0.008
Room temperature *vs*. 37.0°C	ns	ns	0.039
Room temperature *vs*. 38.5°C	ns	0.039	0.010
Between treatments:			
35.5°C vs. 37.0°C	x	ns	ns
37.0°C vs. 38.5°C	ns	ns	0.022
35.5°C vs. 38.5°C	x	0.037	0.016

Pairwise comparisons of fertility (proportion of eggs hatched during 8 weeks of egg production after heat exposure) between females exposed to different heat treatments. The table shows *p* values from paired *t*-tests or Wilcoxon signed rank tests. ns = not significant. x = not tested experimentally.

**Fig 4:**
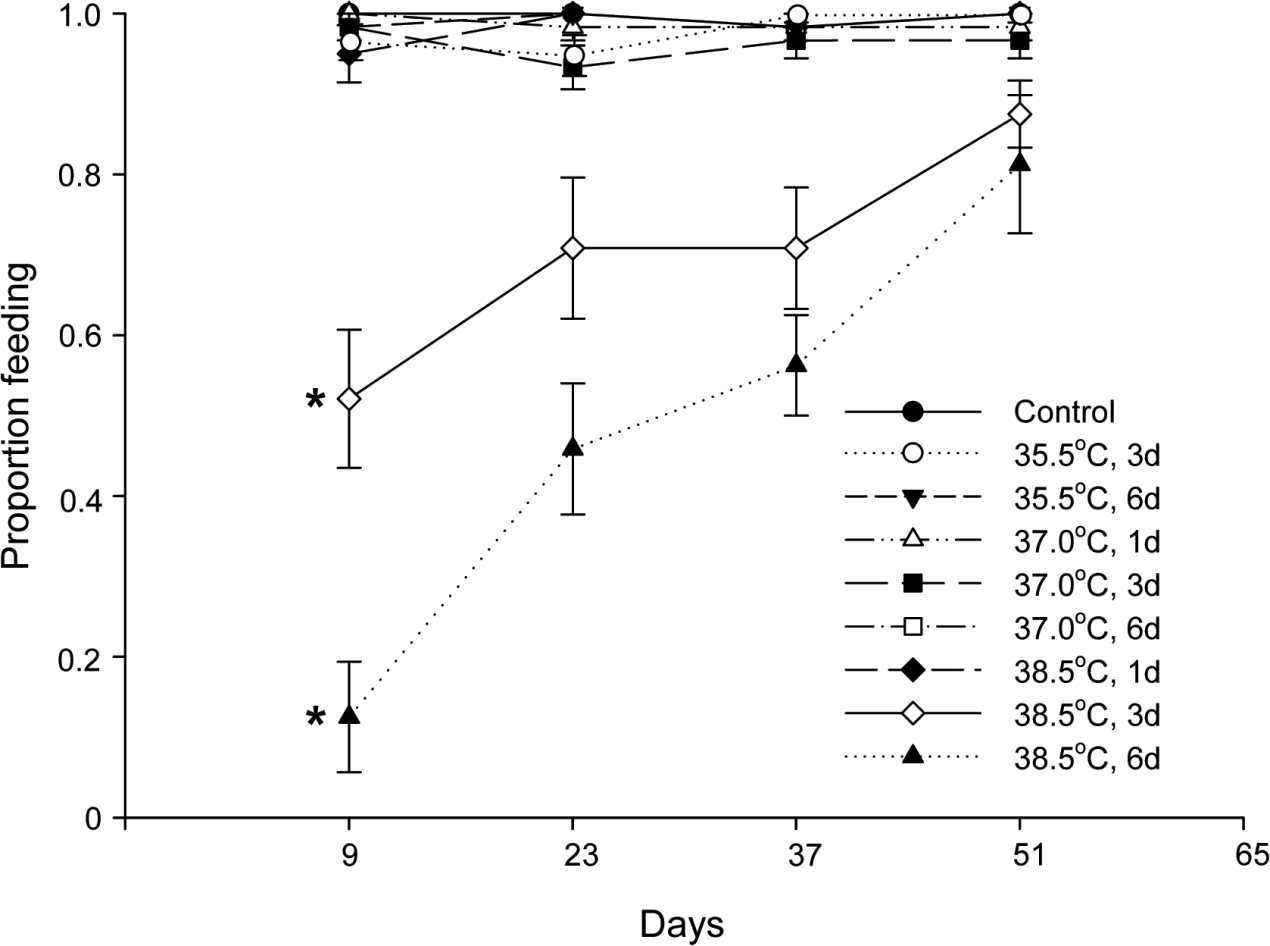
Feeding in heat treated adult *Cimex lectularius*. The proportion of adults feeding (mean ± SE) that had been subjected to different heat treatments is shown. Control = 22.0°C and d = days. * denotes significant difference relative to control (*p* < 0.05).

Bed bugs that experienced 3 and 6 days of exposure to the highest temperature (38.5°C) also suffered from a significantly reduced ability to feed compared with the control (paired *t*-test; 3 days: *t*_3_ = 3.846, *p* = 0.031; 6 days: *t*_3_ = 3.459, *p* = 0.041), whereas the other treatments did not exert such an effect (Wilcoxon signed-rank; 37.0°C for 6 days *vs*. control: *Z* = 1.841, *p* < 0.125 [only the test for the lowest average feeding proportion is shown]; Fig 4). The low proportion of adults that took a blood meal among the affected individuals increased with time after heat treatment and nearly normalized at the fourth feeding.

### Offspring effects

**Fig 5:**
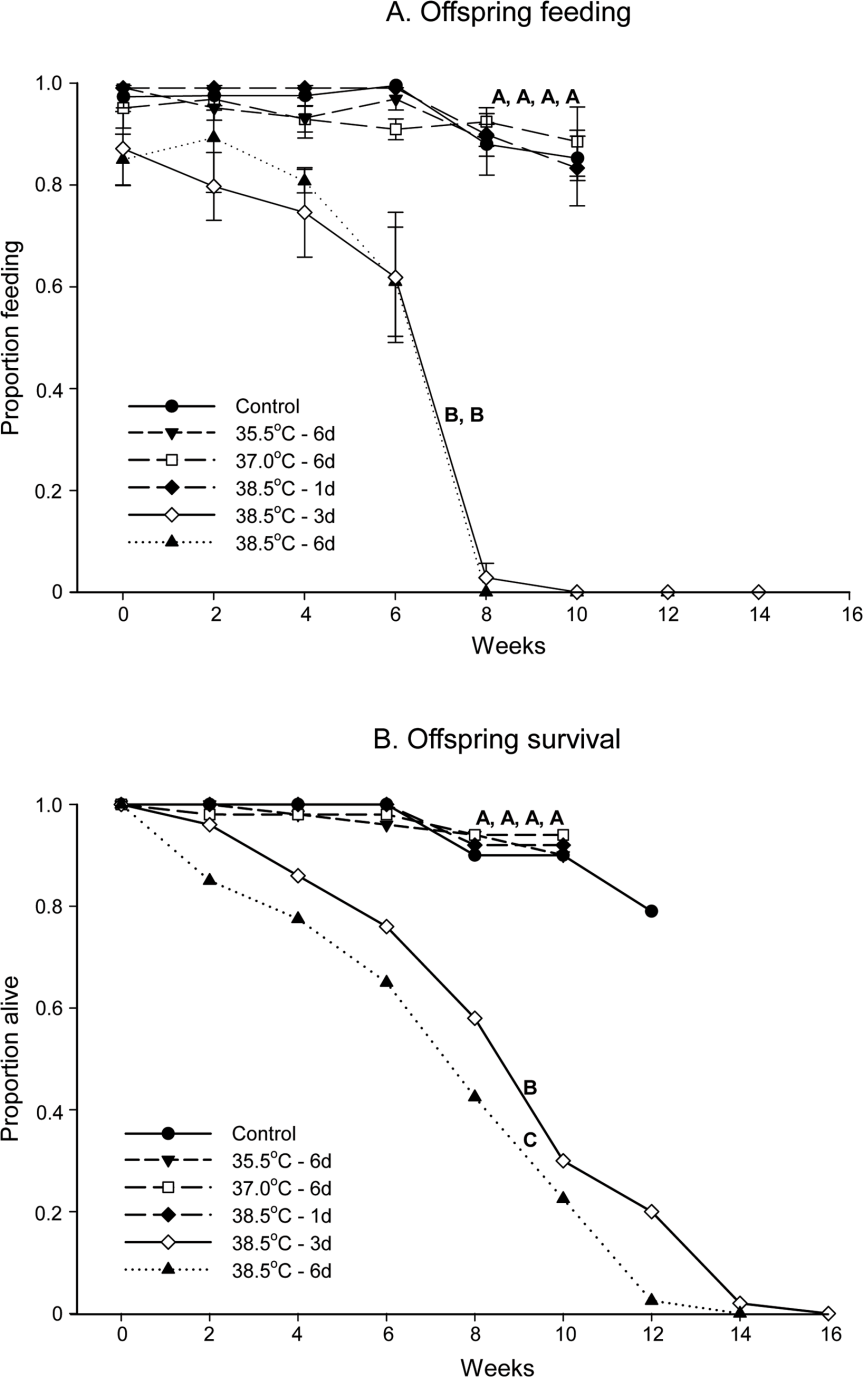
Feeding and survival of *Cimex lectularius* nymphs. The proportion feeding (A) and survival (B) of *Cimex lectularius* nymphs from previously heat treated parents are shown. Each cohort began with 50 nymphs, and all were given the opportunity to feed every 14 days. Feeding (mean ± SE) and survival were recorded until an adult appeared in all boxes in a treatment or all nymphs in a cohort were dead. Control = 22.0°C and d = days. Different letters denote significant differences in feeding or survival between treatments (*p* < 0.05).

Heat treatment of the parents negatively influenced the development of their offspring. This effect was reflected by decreases in feeding, moulting ability, and survival. Offspring that originated from parents that were exposed to 38.5°C for 3 and 6 days exhibited a persistently and significantly reduced ability to feed compared with the control (paired *t*-test; 3 days: *t*_5_ = 3.104, *p* = 0.027; 6 days: *t*_5_ = 2.828, *p* = 0.037). The effect was most evident from the fourth feeding event (Fig 5A). The remaining treatments did not differ significantly from the control in their feeding habits (paired *t*-test; 35.5°C for 6 days: *t*_4_ = 0.667, *p* = 0.541; 37.0°C for 6 days: *t*_5_ = 0.518, *p* = 0.627; 38.5°C for 1 day: *t*_5_ = -1.557, *p* = 0.180).

**Fig 6:**
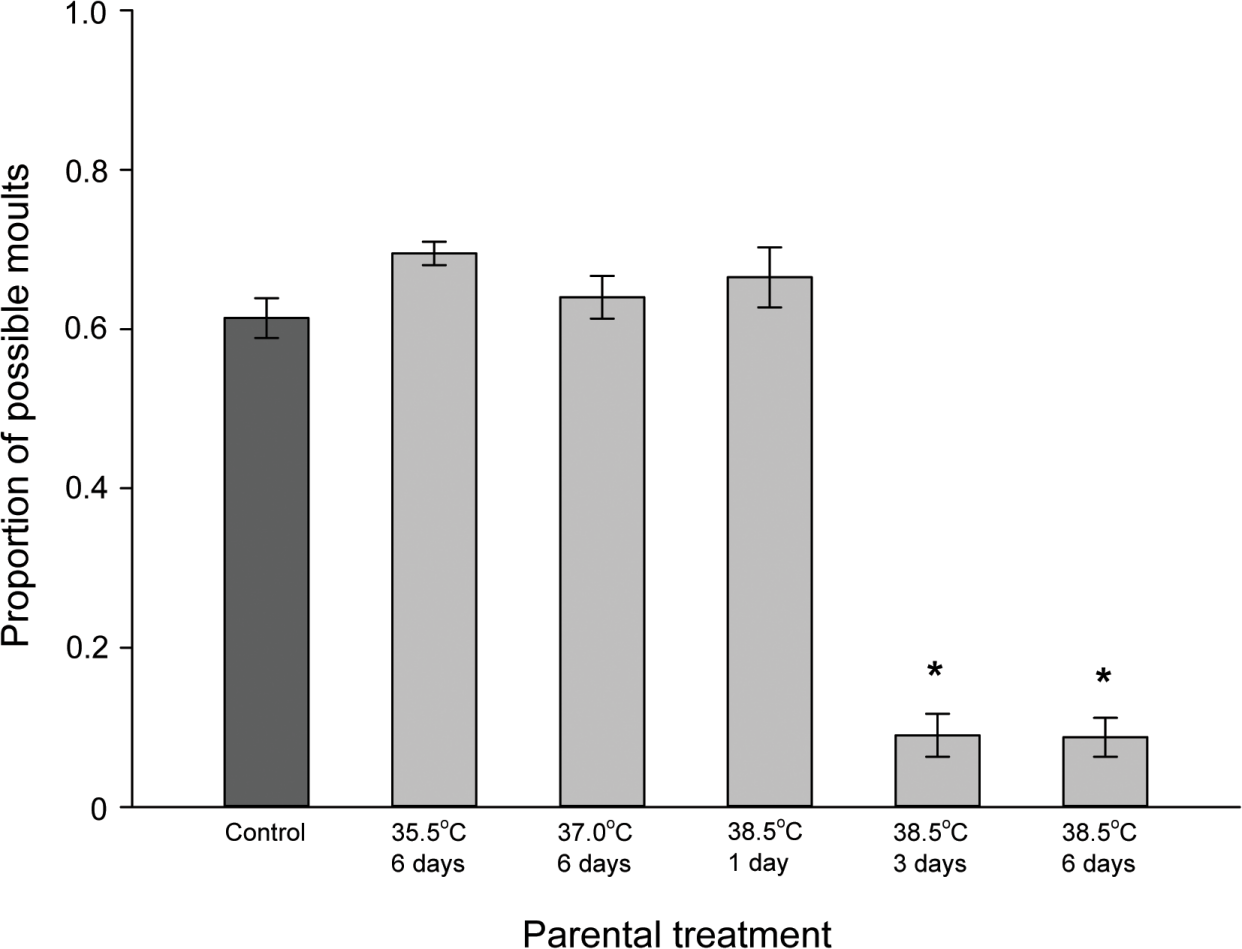
Number of moults completed in *Cimex lectularius* nymphs. The number of moults (mean ± SE) completed after four feeding events by nymphs of parents that had been subjected to different heat treatments is shown. Each cohort began with 50 nymphs, and all were given the opportunity to feed every 14 days. Control = 22.0°C. * denotes significant difference from control (*p* < 0.05).

Nymphs that originated from different heat treatments also differed in the number of moults (ANOVA: *F*_5,24_ = 119.760, *p* < 0.001). Dunnett’s comparison to the control revealed that nymphs that originated from parents that were exposed to 38.5°C for 3 and 6 days exhibited a lower number of moults compared with the control, whereas no such effect was observed with the other treatments (Fig 6). No nymphs from parents that were exposed to 38.5°C for 3 and 6 days, made it past the third nymphal stage, whereas the nymphs from parents that were exposed to the other treatments reached the adult stage after five or six feedings.

Nymphs that originated from parents that were exposed to 38.5°C for 6 days, had lower survival than those that were exposed to the same temperature for 3 days (Kaplan-Meier; *χ* ^*2*^ = 4.51, df = 1, *p* = 0.033; Fig 5B), and both differed significantly from the control and the other treatments (Kaplan-Meier; 38.5°C for 3 days *vs*. 35.5°C for 6 days: *χ* ^*2*^ = 35.67, df = 1, *p* < 0.001, with lower *p* values for the remaining pairwise comparisons).

### Heat treatment of eggs

**Fig 7:**
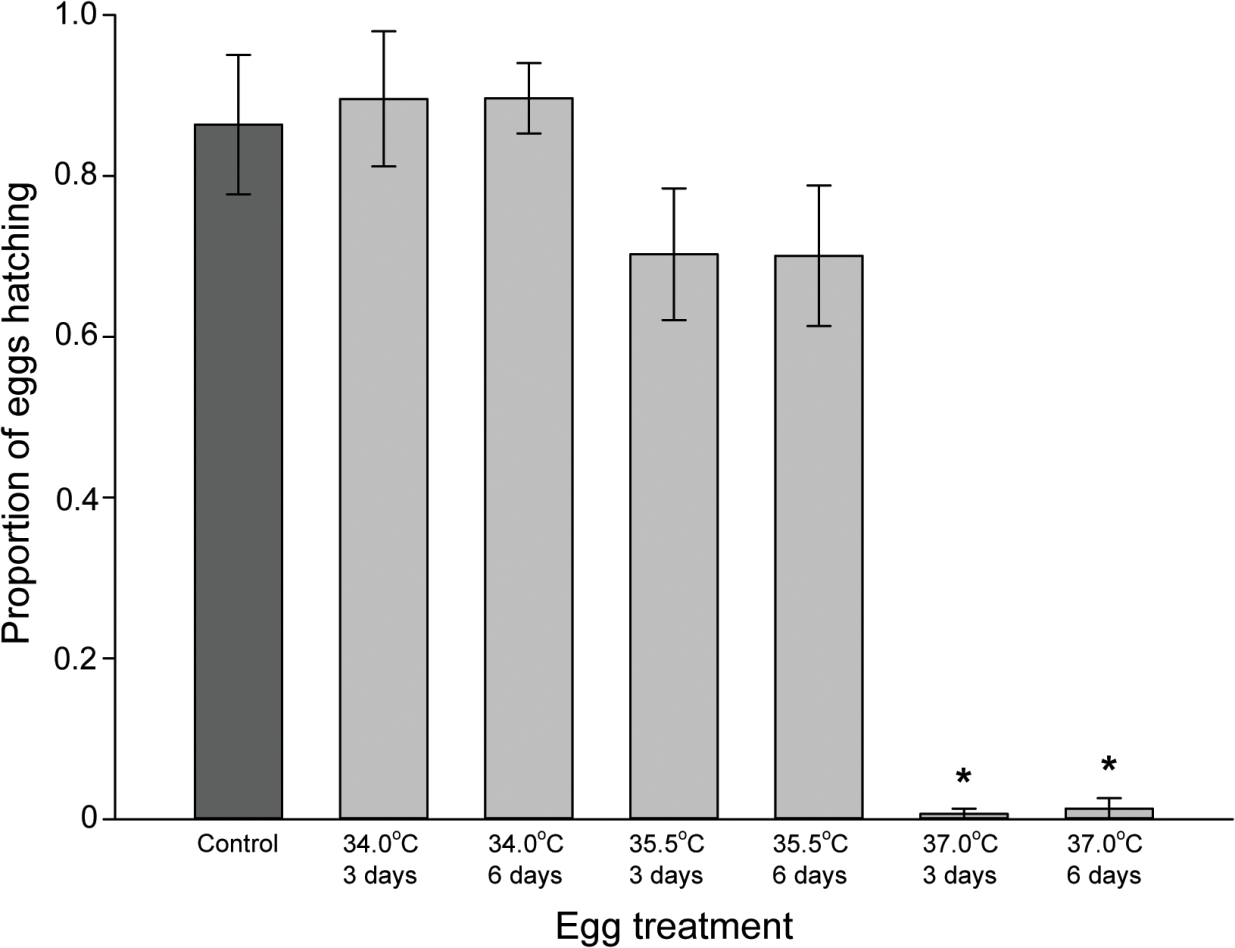
Hatching success of heat treated *Cimex lectularius* eggs. The proportion of *Cimex lectularius* eggs that hatched (mean ± SE) after different heat treatment, is shown. Control = 22.0°C. * denotes significant difference from control (*p* < 0.05).

Heat treatment significantly affected hatching success (Kruskal-Wallis: *H* = 50.507, df = 6, *p* < 0.001; Fig 7), and only 1.0% of the eggs hatched after treatment at 37.0°C. The extended treatment time of 6 days had no apparent additional effect on hatching success at either of the tested temperatures.

## Discussion

The present study showed that sublethal temperatures, well below those that are traditionally used in bed bug control, can be detrimental to essential life history traits. First, adult survival was strongly reduced at temperatures close to 40.0°C for an extended period of time. Second, fecundity was temporarily reduced, and the effect increased with increased temperature and exposure time. Third, the heat treatment of adults also affected their offspring through feeding and moulting arrest, followed by death. Fourth, the eggs showed relatively low heat tolerance when developed and laid under heat stress. These effects may influence total population performance and are all relevant for bed bug establishment, population growth, and recovery after control efforts.

Based on this study and previous reports, there appears to be a critical temperature range between 37.0°C and 40.0°C for bed bugs [23]. We also observed an increase in mortality under moist conditions, but this effect was marginal. The bed bugs in our experimental setting may have experienced a somewhat reduced ability to cool through evaporation under moist conditions [16,18], or they may have suffered more damage through the thermophysical properties of humid air [30]. The time-temperature dependency is clearly important, and even subtle changes of 1.5°C produced large differences in mortality over time. Short-term, manageable temperatures for insects may become lethal if the exposure time is extended [15,31], and sublethal heat stress appears to have the potential to completely knock out or strongly limit bed bug populations. Eggs have previously been found to endure longer than adults when exposed to upper temperature extremes for a short time [10], but our observations indicate that the eggs actually experienced higher mortality than adults at long exposure to sublethal temperatures. High mortality of eggs after prolonged exposure to 35°C—37.0°C has also been previously shown [32,33]. This was confirmed by our study, but we observed egg mortality at even lower temperatures, such as 34.0°C for 9 days, which resulted in hatching success < 50%. We did not investigate the mechanisms that underlie these effects, but the strongly reduced hatching success of eggs deposited at high temperature suggests detrimental effects during oogenesis [34].

The maternal effect that was observed in nymphs with a normal appearance and behaviour, indicates no dysfunction in the insects themselves but instead points to the obligate mutualistic *Wolbachia* bacteria. Bed bugs and *Wolbachia* are integrated into a biological unit, in which the partners are unable to survive independently [35,36]. Many other insects, such as aphids, ants, weevils, and cockroaches, are also associated with bacteria that provide them with essential nutrients [37]. The balance of such associations may be disrupted when the host is exposed to heat stress [38–40]. The complete elimination of these internal symbiotic bacteria is unlikely, but temporary *Wolbachia* knockdown in bed bugs may explain temporary sterilization through a limited supply of essential nutrients that are needed to produce viable eggs [35]. The rebounding of fertility may consequently result from the recovery of the microbial balance in the bacteriomes that normally contain the majority of symbionts. *Wolbachia* is also vertically transmitted from mother to nymphs through oogenesis [36]. If too few symbionts infect the ovaries, for instance following parental heat exposure, then this relationship between generations may be broken. We did not measure the presence of *Wolbachia*, but our findings indicate the involvement of this symbiont. Preventing the transfer across generations may be a key to novel control approaches that target bed bugs. It has also earlier been reported that sublethal heat significantly reduces the reproductive rate of bed bugs and simultaneously renders the mycetomes symbiont-free [41,42]. Even older studies point at potential offspring and population effects of sublethal exposure [32,43,44]. More recently, retarded growth and sterility were found when the *Wolbachia* symbiont was eliminated by antibiotics [36]. This is interesting when considered in light of the present results. If *Wolbachia* can be knocked out with antibiotics and is unable to recover in these crucial organs, then medicinal treatment might be evaluated as a possible bed bug control method to ensure no sustainable population growth. Concerns regarding antibiotic use must be strictly considered, but laboratory population studies are warranted.

The quantification of fecundity and maternal effects is probably the most appropriate response measure for severe conditions, and long-term investigations of sublethal temperatures are essential when considering ecological demands and population-limiting characteristics [15]. As shown herein for bed bugs, the effects of sublethal heat stress might interact with other life history traits, carry over to other stages, or even flow across generations to prolong or enhance the consequences. Mortality is one measurable factor, but progeny success may be equally important in the long term. The temperature-induced sterility lasted for weeks at several of the tested time-temperature combinations, and 3-day exposure to 38.5°C resulted in no viable offspring for more than 2 months. These sterility effects are also likely to combine with general senescence and an age-dependent decline in stress resistance to reduce overall population performance [45–47]. The growth and dispersal potential of bed bugs is tremendous in human habitats, but the effect of a reduced growth rate should not be underestimated [48,49]. In particular not in the fragile situation found during pest control, when the population is targeted with an arsenal of different methods.

Heat treatment targeting temperatures between 45 and 52°C to achieve rapid mortality is a part of commercial integrated pest management approaches against bed bugs [6,10,50]. However, in such treatments temperature may reach 65°C [10] to damage objects and also be highly energy-demanding. The present study indicates that long exposure to sublethal temperatures may be an alternative or supplemental approach. The gap between temperature regimens that are currently used in bed bug control and our experimental approach is large and has both challenges and advantages. One apparent challenge is the necessity of a longer duration of treatment. The present study indicates the lower limits of temperature treatments. The time demand may likely be reduced by elevating temperatures slightly, thus ensuring a more practical approach. The challenge of a long duration may also be met by attaining the desired temperatures in hidden harbourages through the long duration itself and conductive heat transfer within or between objects. A second problem is that both psychological and physiological nuisance must be tolerated by inhabitants for a given time after treatment. This may be solved by properly handling hot spots, such as beds, mattresses, chairs, and sofas, which may easily kill and remove 90–95% of the population [51].

Anecdotal reports also indicate that bed bugs may disperse to other rooms as a result of elevated temperatures, but the precise temperature levels that induce avoidance behaviour have not been thoroughly investigated. How bed bugs behaviourally respond to sublethal heat treatment should be explored to determine the consequences of different heat regimens. Sublethal temperatures may possibly be advantageous by not triggering the escape response, thus ensuring that individuals remain in place for the duration of treatment. Several aspects must be investigated and clarified before proper understanding of sublethal temperature effects can be utilized, but the obvious advantage of lower temperatures is the reduced demand for expensive equipment. The required heating capacity will depend on construction, but in well-insulated bedrooms (which are typical of temperate-to-cold climate areas), 37–38°C temperatures may be easily reached using two to three 1500–2000 W electrical ovens and a fan. The energy cost of a 14-day treatment will consequently not exceed 2000 KWh, thus enabling treatments at an acceptable cost. The use of sublethal temperatures may be included as a part of future multi-method approaches where bed bugs can be targeted by combinations of pesticides, desiccant dusts, essential oils, entomopathogenic fungi, traps, barriers, and alarm- or host signals for activation, [5,52–66]. Support from sublethal heat may contribute to increased mortality and, more importantly, sterilisation, egg mortality, and maternal effects to prevent rebounding populations.

## Conclusions

The present results highlight the importance of life-long measures, including maternal effects when evaluating pest management methods. Short-term investigations may only cover a fraction of the story. Cohort studies and long-term observations may reveal the current and future consequences for population growth. Our study provides more questions than answers, but the management potential of sublethal temperatures should be followed up by studies that investigate temperature regimens that are both practical and suitable for field applications. We only evaluated a few temperature-time combinations. Nothing is known about the potential effects of repeated exposure, strongly fluctuating temperatures, or longer exposure times. Based on the present results, we believe that sublethal heat treatment may be a potential element of future integrated pest management strategies against bed bugs.

## Supporting Information

S1 Dataset Rukke et al 2015. Mortality, temporary sterilization, and maternal effects of sublethal heat in bed bugs.
(XLSX)
